# 2,4-Dichloro-*N*-(3,4-dichloro­phen­yl)benzene­sulfonamide

**DOI:** 10.1107/S1600536809027883

**Published:** 2009-07-22

**Authors:** B. Thimme Gowda, Sabine Foro, P. G. Nirmala, Hartmut Fuess

**Affiliations:** a Department of Chemistry, Mangalore University, Mangalagangotri 574 199, Mangalore, India; b Institute of Materials Science, Darmstadt University of Technology, Petersenstrasse 23, D-64287 Darmstadt, Germany

## Abstract

In the crystal structure of the title compound, C_12_H_7_Cl_4_NO_2_S, the conformation of the N—H bond is *syn* to the *meta*-chloro residue in the aniline benzene ring. The two aromatic rings are tilted relative to each other by 68.9 (1)°. N—H⋯O hydrogen bonds connect the mol­ecules into centrosymmetric dimers.

## Related literature

For related structures, see: Gowda *et al.* (2008 ▶, 2009*a* ▶,*b* ▶). For comparative bond lengths in other aryl sulfonamides, see: Gelbrich *et al.* (2007 ▶); Perlovich *et al.* (2006 ▶).
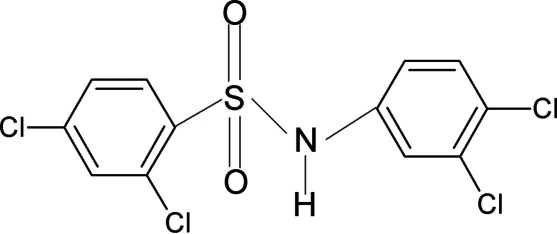


## Experimental

### 

#### Crystal data

C_12_H_7_Cl_4_NO_2_S
*M* *_r_* = 371.05
Triclinic, 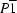

*a* = 8.1498 (9) Å
*b* = 8.2633 (9) Å
*c* = 11.887 (1) Å
α = 81.857 (9)°
β = 72.728 (9)°
γ = 78.213 (9)°
*V* = 745.49 (13) Å^3^
*Z* = 2
Mo *K*α radiation
μ = 0.93 mm^−1^
*T* = 299 K
0.48 × 0.48 × 0.28 mm

#### Data collection

Oxford Diffraction Xcalibur diffractometer with a Sapphire CCD detector
Absorption correction: multi-scan (*CrysAlis RED*; Oxford Diffraction, 2009 ▶) *T*_min_ = 0.664, *T*_max_ = 0.781
7103 measured reflections
2722 independent reflections
2388 reflections with *I* > 2σ(*I*)
*R*_int_ = 0.022

#### Refinement

*R*[*F* ^2^ > 2σ(*F* ^2^)] = 0.044
*wR*(*F* ^2^) = 0.152
*S* = 1.27
2722 reflections
184 parameters
1 restraint
H atoms treated by a mixture of independent and constrained refinement
Δρ_max_ = 0.68 e Å^−3^
Δρ_min_ = −0.48 e Å^−3^

### 

Data collection: *CrysAlis CCD* (Oxford Diffraction, 2009 ▶); cell refinement: *CrysAlis RED* (Oxford Diffraction, 2009 ▶); data reduction: *CrysAlis RED*; program(s) used to solve structure: *SHELXS97* (Sheldrick, 2008 ▶); program(s) used to refine structure: *SHELXL97* (Sheldrick, 2008 ▶); molecular graphics: *PLATON* (Spek, 2009 ▶); software used to prepare material for publication: *SHELXL97*.

## Supplementary Material

Crystal structure: contains datablocks I, global. DOI: 10.1107/S1600536809027883/bt5008sup1.cif

Structure factors: contains datablocks I. DOI: 10.1107/S1600536809027883/bt5008Isup2.hkl

Additional supplementary materials:  crystallographic information; 3D view; checkCIF report

## Figures and Tables

**Table 1:** Hydrogen-bond geometry (Å, °)

*D*—H⋯*A*	*D*—H	H⋯*A*	*D*⋯*A*	*D*—H⋯*A*
N1—H1*N*⋯O1^i^	0.833 (17)	2.079 (18)	2.903 (3)	170 (3)

Symmetry code: (i) 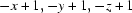.

**Fig. 1.**
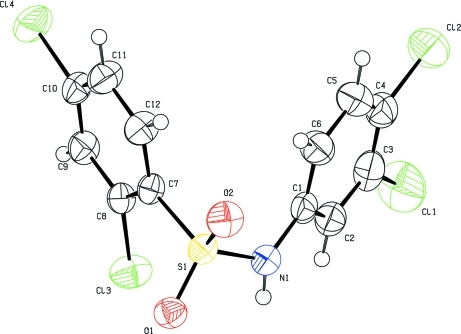
Molecular structure of the title compound, showing the atom labelling scheme and displacement ellipsoids are drawn at the 50% probability level.

**Fig. 2.**
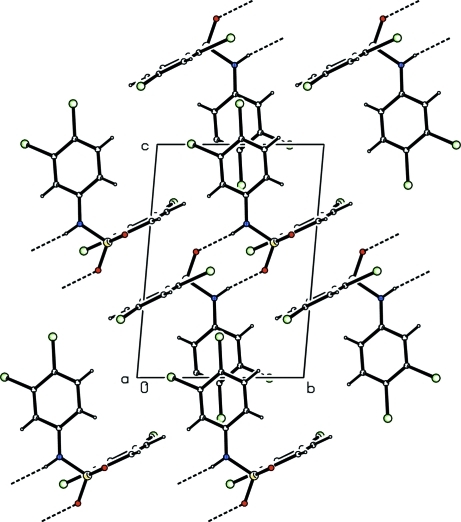
Molecular packing of the title compound with hydrogen bonding shown as dashed lines.

## supplementary crystallographic information

### Comment

In the present work, as part of a study of substituent effects on the structures of *N*-(aryl)-arylsulfonamides  (Gowda *et al.*, 2008; Gowda *et al.*,  2009*a*; Gowda *et al.*, 2009*b*), the structure of 2,4-dichloro-*N*-(3,4-dichlorophenyl)benzenesulfonamide has been determined. The conformations of the N—C bonds in the C—SO_2_—NH—C segment have *trans* and *gauche* torsion angles with the S=O bonds (Fig. 1). The molecule is bent at the S atom with the C—SO_2_—NH—C torsion angle of -48.2 (2)°. The conformation of the N—H bond is *syn* to the *meta*-chloro group in the anilino benzene ring. The two aromatic rings  are tilted relative to each other by 68.9 (1)°. The other bond parameters   are similar to those observed in 2,4-dimethyl-*N*-(phenyl)benzenesulfonamide (Gowda *et al.*, 2009*a*); 4-methyl*N*-(3,4-dimethylphenyl)- benzenesulfonamide (Gowda *et al.*, 2009*b*); *N*-(3-chlorophenyl)benzenesulfonamide (Gowda *et al.*, 2008) and other aryl sulfonamides (Perlovich *et al.*, 2006; Gelbrich *et al.*, 2007). The crystal packing   *via* N—H···O(S) hydrogen bonds (Table 1) is shown in Fig. 2.

### Experimental

The solution of 1,3-dichlorobenzene (10 cc) in chloroform (40 cc) was treated dropwise with chlorosulfonic acid (25 cc) at 0 ° C. After the initial evolution of hydrogen chloride subsided, the reaction mixture was brought to room temperature and poured into crushed ice in a beaker. The chloroform layer was separated, washed with cold water and allowed to evaporate slowly. The residual 2,4-dichlorobenzenesulfonylchloride was treated with 3,4-dichloroaniline in the stoichiometric ratio and boiled for ten minutes. The reaction mixture was then cooled to room temperature and added to ice cold water (100 cc). The resultant solid 2,4-dichloro-*N*-(3,4-dichlorophenyl)benzenesulfonamide was filtered under suction and washed thoroughly with cold water. It was then recrystallized to constant melting point from dilute ethanol. The purity of the compound was checked and characterized by recording its infrared and NMR spectra. The single crystals used in X-ray diffraction studies were grown in ethanolic solution by a slow evaporation at room temperature.

### Refinement

The amino H atom was located in difference map and refined with restrained geometry to 0.86 (2) Å. The other H atoms were positioned with idealized geometry using a riding model with C—H = 0.93 Å. All H atoms were refined with isotropic displacement parameters set to 1.2 times of the *U*_eq_ of the parent atom.

### Crystal data

**Table:**

C_12_H_7_Cl_4_NO_2_S	*Z* = 2
*M**_r_* = 371.05	*F*(000) = 372
Triclinic, *P*1	*D*_x_ = 1.653 Mg m^−^^3^
Hall symbol: -P 1	Mo *K*α radiation, λ = 0.71073 Å
*a* = 8.1498 (9) Å	Cell parameters from 3818 reflections
*b* = 8.2633 (9) Å	θ = 2.5–27.5°
*c* = 11.887 (1) Å	µ = 0.93 mm^−^^1^
α = 81.857 (9)°	*T* = 299 K
β = 72.728 (9)°	Plate, colourless
γ = 78.213 (9)°	0.48 × 0.48 × 0.28 mm
*V* = 745.49 (13) Å^3^	

### Data collection

**Table:**

Oxford Diffraction Xcalibur diffractometer with a Sapphire CCD detector	2722 independent reflections
Radiation source: fine-focus sealed tube	2388 reflections with *I* > 2σ(*I*)
graphite	*R*_int_ = 0.022
Rotation method data acquisition using ω and φ scans	θ_max_ = 25.4°, θ_min_ = 2.5°
Absorption correction: multi-scan (*CrysAlis RED*; Oxford Diffraction, 2009)	*h* = −9→9
*T*_min_ = 0.664, *T*_max_ = 0.781	*k* = −9→9
7103 measured reflections	*l* = −14→14

### Refinement

**Table:**

Refinement on *F*^2^	Primary atom site location: structure-invariant direct methods
Least-squares matrix: full	Secondary atom site location: difference Fourier map
*R*[*F*^2^ > 2σ(*F*^2^)] = 0.044	Hydrogen site location: inferred from neighbouring sites
*wR*(*F*^2^) = 0.152	H atoms treated by a mixture of independent and constrained refinement
*S* = 1.26	*w* = 1/[σ^2^(*F*_o_^2^) + (0.1*P*)^2^] where *P* = (*F*_o_^2^ + 2*F*_c_^2^)/3
2722 reflections	(Δ/σ)_max_ = 0.011
184 parameters	Δρ_max_ = 0.68 e Å^−^^3^
1 restraint	Δρ_min_ = −0.48 e Å^−^^3^

### Special details

**Table:**

Experimental. CrysAlis RED (Oxford Diffraction, 2009) Empirical absorption correction using spherical harmonics, implemented in SCALE3 ABSPACK scaling algorithm.
Geometry. All e.s.d.'s (except the e.s.d. in the dihedral angle between two l.s. planes) are estimated using the full covariance matrix. The cell e.s.d.'s are taken into account individually in the estimation of e.s.d.'s in distances, angles and torsion angles; correlations between e.s.d.'s in cell parameters are only used when they are defined by crystal symmetry. An approximate (isotropic) treatment of cell e.s.d.'s is used for estimating e.s.d.'s involving l.s. planes.
Refinement. Refinement of *F*^2^ against ALL reflections. The weighted *R*-factor *wR* and goodness of fit *S* are based on *F*^2^, conventional *R*-factors *R* are based on *F*, with *F* set to zero for negative *F*^2^. The threshold expression of *F*^2^ > σ(*F*^2^) is used only for calculating *R*-factors(gt) *etc*. and is not relevant to the choice of reflections for refinement. *R*-factors based on *F*^2^ are statistically about twice as large as those based on *F*, and *R*- factors based on ALL data will be even larger.

### Fractional atomic coordinates and isotropic or equivalent isotropic displacement parameters (Å^2^)

**Table:**

	*x*	*y*	*z*	*U*_iso_*/*U*_eq_	
C1	0.3911 (3)	0.5584 (3)	0.7834 (2)	0.0400 (6)	
C2	0.3221 (3)	0.4144 (4)	0.8289 (2)	0.0464 (6)	
H2	0.3248	0.3369	0.7785	0.056*	
C3	0.2503 (3)	0.3869 (4)	0.9484 (3)	0.0551 (7)	
C4	0.2429 (4)	0.5027 (4)	1.0245 (2)	0.0559 (7)	
C5	0.3100 (4)	0.6460 (4)	0.9785 (3)	0.0590 (7)	
H5	0.3034	0.7251	1.0288	0.071*	
C6	0.3868 (4)	0.6734 (4)	0.8589 (2)	0.0509 (7)	
H6	0.4355	0.7685	0.8291	0.061*	
C7	0.2467 (3)	0.8593 (3)	0.61967 (19)	0.0337 (5)	
C8	0.1074 (3)	0.7982 (3)	0.6056 (2)	0.0352 (5)	
C9	−0.0613 (3)	0.8837 (3)	0.6440 (2)	0.0428 (6)	
H9	−0.1543	0.8439	0.6338	0.051*	
C10	−0.0895 (3)	1.0267 (3)	0.6968 (2)	0.0447 (6)	
C11	0.0473 (3)	1.0924 (4)	0.7108 (3)	0.0513 (7)	
H11	0.0262	1.1911	0.7458	0.062*	
C12	0.2136 (3)	1.0071 (3)	0.6715 (2)	0.0448 (6)	
H12	0.3063	1.0493	0.6798	0.054*	
N1	0.4667 (3)	0.5772 (3)	0.65959 (18)	0.0410 (5)	
H1N	0.478 (4)	0.500 (3)	0.618 (2)	0.049*	
O1	0.5157 (2)	0.7111 (2)	0.46042 (14)	0.0419 (4)	
O2	0.5641 (2)	0.8522 (2)	0.61286 (16)	0.0485 (5)	
Cl1	0.16795 (16)	0.20525 (13)	1.00219 (9)	0.0922 (4)	
Cl2	0.15005 (13)	0.47338 (13)	1.17468 (7)	0.0813 (3)	
Cl3	0.13671 (8)	0.61471 (9)	0.54309 (6)	0.0544 (3)	
Cl4	−0.30088 (9)	1.12858 (12)	0.75011 (8)	0.0705 (3)	
S1	0.46469 (7)	0.75391 (7)	0.57981 (5)	0.0351 (2)	

### Atomic displacement parameters (Å^2^)

**Table:**

	*U*^11^	*U*^22^	*U*^33^	*U*^12^	*U*^13^	*U*^23^
C1	0.0316 (12)	0.0451 (14)	0.0404 (12)	0.0022 (10)	−0.0130 (9)	−0.0006 (10)
C2	0.0439 (14)	0.0462 (15)	0.0468 (14)	−0.0028 (12)	−0.0137 (11)	−0.0012 (12)
C3	0.0428 (15)	0.0574 (18)	0.0573 (16)	−0.0035 (13)	−0.0135 (12)	0.0126 (14)
C4	0.0497 (16)	0.063 (2)	0.0445 (14)	0.0031 (14)	−0.0093 (12)	0.0021 (13)
C5	0.0676 (19)	0.0600 (19)	0.0475 (16)	−0.0058 (15)	−0.0149 (14)	−0.0083 (14)
C6	0.0569 (16)	0.0503 (17)	0.0459 (14)	−0.0101 (13)	−0.0143 (12)	−0.0040 (12)
C7	0.0279 (11)	0.0346 (12)	0.0387 (11)	−0.0069 (9)	−0.0102 (9)	0.0007 (9)
C8	0.0336 (12)	0.0363 (13)	0.0376 (11)	−0.0085 (10)	−0.0121 (9)	−0.0012 (9)
C9	0.0296 (12)	0.0553 (17)	0.0459 (13)	−0.0081 (11)	−0.0133 (10)	−0.0050 (12)
C10	0.0344 (12)	0.0496 (15)	0.0466 (13)	0.0040 (11)	−0.0136 (10)	−0.0041 (11)
C11	0.0471 (15)	0.0437 (15)	0.0649 (17)	0.0001 (12)	−0.0191 (13)	−0.0141 (13)
C12	0.0394 (14)	0.0414 (14)	0.0582 (15)	−0.0095 (11)	−0.0165 (11)	−0.0089 (12)
N1	0.0415 (11)	0.0400 (12)	0.0400 (11)	−0.0023 (9)	−0.0121 (9)	−0.0036 (9)
O1	0.0400 (9)	0.0414 (10)	0.0410 (9)	−0.0075 (7)	−0.0059 (7)	−0.0036 (7)
O2	0.0312 (9)	0.0583 (12)	0.0615 (11)	−0.0144 (8)	−0.0142 (8)	−0.0106 (9)
Cl1	0.1158 (8)	0.0755 (7)	0.0797 (7)	−0.0406 (6)	−0.0147 (6)	0.0204 (5)
Cl2	0.0879 (6)	0.0890 (7)	0.0450 (4)	−0.0018 (5)	0.0006 (4)	0.0053 (4)
Cl3	0.0432 (4)	0.0551 (5)	0.0720 (5)	−0.0139 (3)	−0.0140 (3)	−0.0250 (4)
Cl4	0.0396 (4)	0.0852 (6)	0.0818 (6)	0.0187 (4)	−0.0207 (4)	−0.0253 (5)
S1	0.0265 (3)	0.0384 (4)	0.0406 (4)	−0.0070 (3)	−0.0087 (2)	−0.0033 (3)

### Geometric parameters (Å, °)

**Table:**

C1—C6	1.386 (4)	C7—S1	1.768 (2)
C1—C2	1.391 (4)	C8—C9	1.385 (3)
C1—N1	1.416 (3)	C8—Cl3	1.724 (2)
C2—C3	1.372 (4)	C9—C10	1.363 (4)
C2—H2	0.9300	C9—H9	0.9300
C3—C4	1.388 (4)	C10—C11	1.396 (4)
C3—Cl1	1.731 (3)	C10—Cl4	1.729 (3)
C4—C5	1.380 (4)	C11—C12	1.371 (4)
C4—Cl2	1.723 (3)	C11—H11	0.9300
C5—C6	1.380 (4)	C12—H12	0.9300
C5—H5	0.9300	N1—S1	1.625 (2)
C6—H6	0.9300	N1—H1N	0.833 (17)
C7—C12	1.387 (3)	O1—S1	1.4297 (17)
C7—C8	1.394 (3)	O2—S1	1.4208 (16)
			
C6—C1—C2	119.9 (2)	C7—C8—Cl3	121.98 (19)
C6—C1—N1	123.2 (2)	C10—C9—C8	119.3 (2)
C2—C1—N1	116.9 (2)	C10—C9—H9	120.3
C3—C2—C1	119.9 (3)	C8—C9—H9	120.3
C3—C2—H2	120.1	C9—C10—C11	121.9 (2)
C1—C2—H2	120.1	C9—C10—Cl4	119.22 (19)
C2—C3—C4	120.7 (3)	C11—C10—Cl4	118.8 (2)
C2—C3—Cl1	118.6 (2)	C12—C11—C10	118.0 (3)
C4—C3—Cl1	120.7 (2)	C12—C11—H11	121.0
C5—C4—C3	119.2 (3)	C10—C11—H11	121.0
C5—C4—Cl2	119.1 (2)	C11—C12—C7	121.5 (2)
C3—C4—Cl2	121.8 (2)	C11—C12—H12	119.2
C6—C5—C4	120.8 (3)	C7—C12—H12	119.2
C6—C5—H5	119.6	C1—N1—S1	124.52 (17)
C4—C5—H5	119.6	C1—N1—H1N	121 (2)
C5—C6—C1	119.6 (3)	S1—N1—H1N	110 (2)
C5—C6—H6	120.2	O2—S1—O1	118.63 (10)
C1—C6—H6	120.2	O2—S1—N1	109.79 (11)
C12—C7—C8	119.0 (2)	O1—S1—N1	104.74 (10)
C12—C7—S1	117.67 (17)	O2—S1—C7	105.89 (11)
C8—C7—S1	123.32 (18)	O1—S1—C7	111.08 (10)
C9—C8—C7	120.2 (2)	N1—S1—C7	106.12 (11)
C9—C8—Cl3	117.83 (17)		
			
C6—C1—C2—C3	0.0 (4)	C8—C9—C10—C11	1.7 (4)
N1—C1—C2—C3	178.6 (2)	C8—C9—C10—Cl4	−177.29 (18)
C1—C2—C3—C4	1.1 (4)	C9—C10—C11—C12	−1.1 (4)
C1—C2—C3—Cl1	−179.28 (19)	Cl4—C10—C11—C12	177.9 (2)
C2—C3—C4—C5	−0.4 (4)	C10—C11—C12—C7	−0.3 (4)
Cl1—C3—C4—C5	179.9 (2)	C8—C7—C12—C11	1.1 (4)
C2—C3—C4—Cl2	178.9 (2)	S1—C7—C12—C11	−176.5 (2)
Cl1—C3—C4—Cl2	−0.8 (4)	C6—C1—N1—S1	−34.0 (3)
C3—C4—C5—C6	−1.3 (5)	C2—C1—N1—S1	147.43 (19)
Cl2—C4—C5—C6	179.4 (2)	C1—N1—S1—O2	65.2 (2)
C4—C5—C6—C1	2.3 (5)	C1—N1—S1—O1	−166.37 (17)
C2—C1—C6—C5	−1.7 (4)	C1—N1—S1—C7	−48.8 (2)
N1—C1—C6—C5	179.8 (2)	C12—C7—S1—O2	0.2 (2)
C12—C7—C8—C9	−0.5 (4)	C8—C7—S1—O2	−177.34 (19)
S1—C7—C8—C9	176.96 (18)	C12—C7—S1—O1	−129.9 (2)
C12—C7—C8—Cl3	−179.53 (19)	C8—C7—S1—O1	52.6 (2)
S1—C7—C8—Cl3	−2.0 (3)	C12—C7—S1—N1	116.8 (2)
C7—C8—C9—C10	−0.8 (4)	C8—C7—S1—N1	−60.7 (2)
Cl3—C8—C9—C10	178.2 (2)		

### Hydrogen-bond geometry (Å, °)

**Table:**

*D*—H···*A*	*D*—H	H···*A*	*D*···*A*	*D*—H···*A*
N1—H1N···O1^i^	0.83 (2)	2.08 (2)	2.903 (3)	170 (3)

Symmetry codes: (i) −*x*+1, −*y*+1, −*z*+1.
